# Resveratrol-Enriched Rice Attenuates UVB-ROS-Induced Skin Aging via Downregulation of Inflammatory Cascades

**DOI:** 10.1155/2017/8379539

**Published:** 2017-08-16

**Authors:** Lalita Subedi, Taek Hwan Lee, Hussain Mustatab Wahedi, So-Hyeon Baek, Sun Yeou Kim

**Affiliations:** ^1^Laboratory of Pharmacognosy, College of Pharmacy and Gachon Institute of Pharmaceutical Sciences, Gachon University, Incheon 21936, Republic of Korea; ^2^College of Pharmacy, Yonsei University, No. 162-1, Songdo-dong, Yeonsu-gu, Incheon 406-840, Republic of Korea; ^3^Department of Biochemistry, Faculty of Biological Sciences, Quaid-i-Azam University, Islamabad, Pakistan; ^4^National Institute of Crop Science, Rural Development Administration, Iksan 570-080, Republic of Korea

## Abstract

The skin is the outermost protective barrier between the internal and external environments in humans. Chronic exposure to ultraviolet (UV) radiation is a major cause of skin aging. UVB radiation penetrates the skin and induces ROS production that activates three major skin aging cascades: matrix metalloproteinase- (MMP-) 1-mediated aging; MAPK-AP-1/NF-*κ*B-TNF-*α*/IL-6, iNOS, and COX-2-mediated inflammation-induced aging; and p53-Bax-cleaved caspase-3-cytochrome C-mediated apoptosis-induced aging. These mechanisms are collectively responsible for the wrinkling and photoaging characteristic of UVB-induced skin aging. There is an urgent requirement for a treatment that not only controls these pathways to prevent skin aging but also avoids the adverse effects often encountered when applying bioactive compounds in concentrated doses. In this study, we investigated the efficacy of genetically modified normal edible rice (NR) that produces the antiaging compound resveratrol (R) as a treatment for skin aging. This resveratrol-enriched rice (RR) overcomes the drawbacks of R and enhances its antiaging potential by controlling the abovementioned three major pathways of skin aging. RR does not exhibit the toxicity of R alone and promisingly downregulates the pathways underlying UVB-ROS-induced skin aging. These findings advocate the use of RR as a nutraceutical for antiaging purposes.

## 1. Introduction

In humans, the skin is the outermost barrier between the internal and external environments [1]. Internal factors, such as genetic changes, can cause intrinsic aging while external factors, such as UVB and environmental toxins, can result in extrinsic aging [2]. Long-term exposure to ultraviolet (UV) radiation is a major cause of skin aging [3]. Histologically, wrinkled skin is characterized by the accumulation of altered elastic fibers and degradation or degeneration of collagen bundles in the dermis [4, 5]. UVB-induced ROS production activates mitogen-activated protein kinase (MAPK) signaling and the transcription factors activator protein-1 (AP-1) and nuclear factor-*κ*B (NF-*κ*B), which further induce the inflammaging and apoptosis in cells and cause skin aging.

UVB can induce an imbalance in mitochondrial fusion and fission that itself causes mitochondrial dysfunction, oxidative stress, prolonged inflammation, and increased apoptosis, which are the major hallmarks of skin aging [6]. Hence, the mechanisms underlying skin photoaging and wrinkling are closely associated with the inflammaging, apoptosis, and ROS-induced damage that occurs as part of the normal homeostatic processes in the skin [7, 8–11]. Aging-associated inflammation, otherwise known as inflammaging, is a major consequence of immunosenescence. Most aging-associated diseases share inflammation-related characteristics, such as upregulated tumor necrosis factor-*α* (TNF-*α*) and interleukin-6 (IL-6) levels, which further complicate such conditions [12]. Inflammaging is responsible for the activation of transcription factors such as NF-*κ*B and sirtuins, which propagate inflammation-induced signals and aggravate skin aging by inducing apoptosis and increased ROS production [13]. The production of ROS and matrix metalloproteinases is common in both intrinsic and extrinsic aging. It has been reported that the accumulation of ROS induces the activation of MAPK pathways. The activation of extracellular signal-regulated kinases (ERKs), c-Jun N-terminal kinases (JNKs), and p38 MAPKs induces the activation of the transcription factors AP-1 and NF-*κ*B. Additionally, there is upregulated transcription of inflammatory mediators such as NO, iNOS, COX-2, and proinflammatory cytokines (including TNF-*α* and IL-6) [14]. Such inflammatory mediators will further induce collagen degradation by promoting apoptosis in dermal fibroblasts, enhancing the expression of the matrix metalloproteinases MMP-1, MMP-3, and MMP-9, and preventing the expression of procollagen [15]. In particular, UVB- or ROS-induced MMP-1, known as interstitial collagenase, initiates the degradation of TGF-*β*, elastin, and collagen types I, II, and III, especially procollagen type I (PIP-1) [16, 17]. These cascades have also been shown to induce inflammation and apoptosis in cultured cells, further hastening the skin aging process [18]. Apoptosis can result from direct DNA damage (intrinsic), the clustering of death receptors on the cell surface (extrinsic), and the generation of ROS and activation of tumor suppressor gene p53-mediated modulation of Bcl2 family proteins [19].

Natural products are often used in the cosmetics industry because of the consumers' growing preference for environment-friendly items [20]. Resveratrol (R) is a trihydroxy derivative of stilbene (3,5,4′-trihydroxystilbene) that is present in grapes, berries, peanuts, and red wine [21]. It has been widely used in the cosmetics and pharmaceutical industries for its antitumor, anti-inflammatory, antiaging, and antimelanogenic effects [21–23]. R is particularly well suited to addressing inflammatory processes in the skin, because its antioxidant properties work well against the high levels of oxidative stress frequently encountered by skin cells. However, there are several impediments to applying this promising agent as a treatment, such as its poor bioavailability and fast metabolism [24]. Most of the adverse effects associated with R occur at higher doses and relate primarily to nephrotoxicity. Such limitations have attracted attention from researchers seeking to design a derivative, nanoparticle, or genetically engineered vehicle for R, so that its therapeutic effect can be elicited in a safer, more effective, and more promising way. In the case of designing a genetically engineered vehicle for R, it is necessary to select a foodstuff that can be easily consumed or applied. These conditions led us to consider using rice (*Oryza sativa* L. var. *japonica*) as the vehicle for a genetically engineered R product. Rice has been used in folk medicine and the cosmetics industry in Korea, China, and Japan for many years [25]. It has been used for the treatment of various allergic disorders, such as dermatitis and bronchitis, as well as skin aging and other conditions [26–28]. Because of the demonstrated biological efficacy of R and normal rice (NR) in various skin disorders, we hypothesized that resveratrol-enriched rice (RR) may exhibit synergistic or additive effects. The transgenic cereal crop, called RR, was designed to overexpress the stilbene synthase gene isolated from the peanut (*Arachis hypogaea* var. Palkwang) and therefore contains high levels of R. The excellent antiobesity and antimelanogenic effects of RR have previously been demonstrated [29]. We have previously reported that the effects of NR and R combine synergistically in RR, when used to treat obesity in mice fed on a high-fat diet or to control metabolic syndrome and its related disorders [29, 30]. However, the efficacy of RR in a UVB-induced skin aging model has not been reported thus far. Hence, we investigated the antiwrinkle properties of RR relative to those of R or NR. In order to evaluate the antiwrinkle properties of RR, we used a cellular model of photoaging (UVB-induced damage to dermal fibroblasts) and determined the effects of RR, R, and NR on aging-related parameters.

The search for improved cosmetic products has prompted the development of multifunctional cosmetic formulations. Those formulations that harness the synergistic effects of different active substances and maintain integrity against UVB-induced toxicity could be better candidates for the prevention and treatment of UVB-induced skin aging and skin disorders. Three of the major molecular pathways that can be downregulated to reduce skin aging (characterized by skin wrinkles and photoaging) include MMP-mediated aging, inflammaging, and apoptosis-induced aging. This study demonstrated that NR, R, and RR have good potential for protecting the skin against UVB-induced toxicity. The additive effect elicited by RR renders it a potential candidate for the preparation of safe and effective cosmeceuticals in the future. This study found that genetically engineered natural products can not only be better for skin protection but also safer and of greater potential utility as a cosmetic preparation.

## 2. Methods

### 2.1. Materials and Chemicals

Dulbecco's modified eagle's medium (DMEM), fetal bovine serum (FBS), and penicillin streptomycin were purchased from Gibco BRL (Grand Island, NY, USA). Dimethyl sulfoxide (DMSO) and 3-(4,5-dimethylthiazol-2-yl)-2,5-diphenyltetrazolium bromide (MTT) were purchased from Sigma-Aldrich (St. Louis, MO, USA). An enzyme-linked immunosorbent assay (ELISA) kit for PIP-1 was obtained from Takara (Procollagen Type I C-Peptide enzyme immunoassay (EIA) Kit; Takara, Shiga, Japan). The ELISA kit for MMP-1, TNF-*α*, and IL-6 was purchased from R&D Systems (Human Total MMP-1, TNF-*α*, and IL-6 kit R&D Systems Inc., Minneapolis, MN, USA). Transfer membrane was purchased from the Millipore Corporation (Bedford, MA, USA). Materials for the enhanced chemiluminescence (ECL) detection and lysis buffer for skin cells and tissues were purchased from Intron (Sungnam, Korea). The antibodies against *α*-tubulin, MMP-1, type I procollagen, iNOS, COX-2, ERK, JNK, p38, Bax, Bcl2, cleaved caspase-3, p53, TGF-*β*, and elastin were purchased from Santa Cruz (Dallas, TX, USA), Cell Science (Canton, MA, USA), and Cell Signaling (Beverly, MA, USA). Secondary antibodies conjugated to horseradish peroxidase were purchased from Santa Cruz. Resveratrol was purchased from Sigma-Aldrich (St. Louis, Missouri, USA). Rice (NR) (*Oryza sativa* var. japonica) and resveratrol-enriched rice (RR) were supplied by the Rural Development Administration (RDA) of South Korea.

### 2.2. Extract Preparation

NR and RR obtained from RDA were undergone for extraction in methanol. Firstly, each sample weighed 10 g. 100 mL MeOH was added in both crude drugs and then placed in an ultrasonic bath for 60 min with sonication. After 60 min incubation for extraction, the mixture was filtered and evaporated using rotary evaporator followed by freeze drying for complete evaporation. The obtained yield was dissolved in MeOH in order to make a stock of 10 mg/mL concentration. This stock was diluted and used for the treatment of cells as well as reconstructed skin tissue during experiment.

### 2.3. Cell Culturing

Normal human dermal fibroblast cells (NHDFs) were obtained by skin biopsy from a healthy young male donor (MCTT Core Inc., Seoul, Korea). The cells were plated in 100 mm tissue culture dishes and cultured in DMEM supplemented with 10% heat-inactivated FBS and 1% penicillin-streptomycin at 37°C in a humidified atmosphere with 5% CO_2_. Cells were cultured in 100 mm culture dishes and seeded in 60 mm culture dishes (1.2 × 10^5^ cells/well) when they reached more than 80% confluence. All experiments were performed using cells between passages 6 and 10.

### 2.4. UVB Irradiation and Sample Treatments

UVB irradiation and treatment with the samples were performed according to a method previously reported by Hwang et al. [5]. When NHDFs seeded in 60 mm culture dishes covered more than 80% of the dish, the cells were washed twice with phosphate-buffered saline (PBS). The cells were suspended in a small amount of PBS and exposed to UVB (144 mJ/cm^2^) using a UVB irradiation machine (Bio-Link BLX-312; Vilber Lourmat GmbH, Marne-la-Vallée, France). After UVB irradiation, the cells were washed with warm PBS three times. The cells were immediately treated with the samples NR, RR, and R (10 and 100 *μ*g/mL) under serum-free medium conditions. Nonirradiated control cells were maintained under the same culture conditions without UVB exposure.

### 2.5. Measurement of Cell Viability (MTT Assay)

The MTT assay measures cell viability by monitoring color change during the reduction of MTT to formazan dye, which is purple in color. MTT assay was performed as described previously [31] with slight modification. NHDF cells were treated with UVB followed by sample treatment and incubated for total 72 h. After 72 h of incubation, the volume of the medium was reduced to 1 mL, and 100 *μ*L of 1 mg/mL MTT was added to each well. Next, the cells were incubated in the presence of 5% CO_2_ at 37°C for 2 h. The substrate-containing medium was removed, and 800 *μ*L of DMSO was added to each well to dissolve the formazan crystals. The plates were shaken on an orbital shaker for 10 min at room temperature. The absorbance of 100 *μ*L aliquots of formazan dissolved in DMSO was quantified by measuring the optical density (OD) at 570 nm using an ELISA reader (Molecular Devices E09090; San Francisco, CA, USA).

### 2.6. Measurement of ROS Production

After 24 h of UVB irradiation (144 mJ/cm^2^) and sample treatment, NHDFs were stained with 30 *μ*M 2′,7′-dichlorofluorescein diacetate (DCFH-DA; Sigma-Aldrich) for 30 min at 37°C in a CO_2_ incubator. The cells were then analyzed using flow cytometry (FACSCalibur™; Becton-Dickinson, San Jose, CA, USA).

### 2.7. Measurement of MMP-1, Type I Procollagen, TNF-*α*, and IL-6

After 72 h of incubation, cell medium was collected from each well. The concentrations of MMP-1, type I procollagen, TNF-*α*, and IL-6 were analyzed from conditioned medium using commercially available ELISA kits (Human Total MMP-1, TNF-*α*, and IL-6 kit; R&D Systems Inc.; Procollagen Type I C-Peptide EIA Kit, Takara) in accordance with the manufacturers' instructions. Each sample was analyzed in triplicate.

### 2.8. Western Blot Analysis

For the Western blot analysis, cells were lysed with lysis buffer (50 mM Tris-Cl, pH 8.0, 0.1% sodium dodecyl sulfate (SDS), 150 mM NaCl, 1% NP-40, 0.02% sodium azide, 0.5% sodium deoxycholate, 100 *μ*g/mL phenylmethylsulfonyl fluoride, 1 *μ*g/mL aprotinin, and phosphatase inhibitor) and centrifuged at 12,000 ×g for 20 min at 4°C temperature. Cell and skin lysates were then homogenized to yield equivalent amounts of protein based on protein concentration measurements carried out with Bradford reagent (Bio-Rad, Hercules, CA, USA). Homogenized proteins were resolved using 6% or 10% SDS polyacrylamide gel electrophoresis (SDS-PAGE) and transferred to nitrocellulose membranes (Amersham Pharmacia Biotech, Buckinghamshire, UK). The membranes were then blocked with 5% nonfat milk in Tris-buffered saline with tween (TBST) (50 mmol/L Tris-HCl, pH 7.5, 150 mmol/L NaCl, and 0.1% Tween 20) for 1 h at room temperature to block nonspecific interactions. The membranes were incubated in primary antibodies overnight at 4°C, washed with TBST three times, and incubated with secondary antibody (Santa Cruz Biotechnology Inc.) for 1 h at room temperature. Protein levels were determined using ECL reagents (Fujifilm, LAS-4000, Tokyo, Japan) and Image Master TM 17 2D Elite software, version 3.1 (Amersham Pharmacia Biotech, NJ, USA).

### 2.9. Reconstructed Human Skin Tissue Model

Reconstructed human skin (Keraskin™ FT) was purchased from Modern Cell & Tissue Technologies Inc. (Seoul, Korea). The reconstructed skin model is composed of multilayered keratinocytes and fibroblasts. To evaluate the effects of NR, RR, and R on UVB-induced photoaging, the reconstructed skin was topically treated with the samples. The samples were dissolved at a concentration of 1% (*w/v*) in 10% propylene glycol with phosphate-buffered saline (PBS) to form the treatment solution, 20 *μ*L of which was applied to the reconstructed skin. After 24 h, the skin tissue was exposed to 100 mJ/cm^2^ UVB radiation. The UV source, which generated radiation at a wavelength of 310 nm, was supplied by Sankyo Denki sunlamps (Kanagawa, Japan). After 24 h, the skin tissue was collected and fixed in 10% formalin and processed for histological analysis. Paraffin sections (4 *μ*m) were stained with hematoxylin-eosin (H&E) and Masson's trichrome (MT) and immunohistochemically analyzed. To carry out the immunohistochemical analysis, the sections were incubated in 0.1% protease in PBS for antigen retrieval and were then incubated in 3% H_2_O_2_ in PBS for 10–15 min. The sections were incubated with 2% normal horse serum in PBS. After 1 h, the sections were incubated with primary antibody procollagen type I (Santa Cruz Biotechnology Inc.) and MMP-1 (Abcam, Cambridge, MA, USA). After washing with PBS, the slides were incubated in Vectastain ABC reagent (Vector Laboratory, Piscataway, NJ, USA) for 1 h. The color was developed with 3,3′-diaminobenzidine (DAB).

### 2.10. Statistical Analysis

The results were evaluated using the Statistical Analysis System (GraphPad Prism 5, La Jolla, CA, USA). The results are presented as mean ± standard error of the mean (SEM), and all results are the mean of at least three independent experiments. A statistical comparison of different treatment groups was determined by one-way analysis of variance (ANOVA) followed by Newman-Keuls multiple comparison test. A value of *p* < 0.05 was considered statistically significant.

## 3. Results

### 3.1. RR Protects against UVB-Induced Toxicity in NHDF Cells

**Figure 1:**
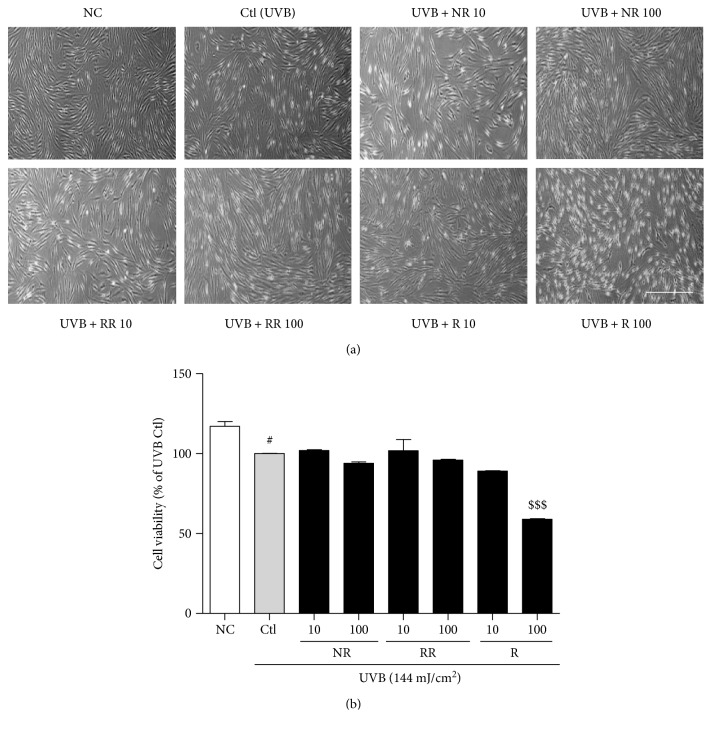
Normal human dermal fibroblast (NHDF). (a) Cell morphology and (b) cell viability after 72 h of treatment with or without 144 mJ/cm^2^ ultraviolet B (UVB) and 10 or 100 *μ*g/mL of the following samples: normal rice (NR), resveratrol-enriched rice (RR), and resveratrol (R). All data are presented as the mean ± SEM of three independent experiments. ^#^*p* < 0.05 versus the normal control. ^$$$^*p* < 0.001 indicates the significant toxicity versus a UVB-irradiated control. NC is normal control, Ctl is UVB control, NR is normal rice, RR is resveratrol-enriched rice, and R is resveratrol. NR and RR were treated in *μ*g/mL, and R was treated in *μ*M.

UVB exposure induces cell death in dermal fibroblasts, as well as various inflammatory cascades, resulting in skin aging, skin wrinkling, and skin pigmentation. In order to evaluate the cytotoxicity of the samples, after 72 h of UVB (144 mJ/cm^2^) and sample treatment, the viability of NHDF cells was measured using an MTT assay (as described in the Methods). We photographed the cells to show their morphology with or without UVB and sample treatment. In the UVB-exposed cells, NR and RR were not toxic and did not affect the normal morphology of NHDF cells until the concentration reached 100 *μ*g/mL. However, R alone showed significant toxicity, causing cell death at the higher concentration of 100 *μ*g/mL and a completely altered, shrunken cell morphology. Using a lower concentration (10 *μ*g/mL) of R induced the level of cell death, but its significant toxicity was still revealed at this dose by comparing the morphology of the treated cells with that of the cells in the UVB-treated control group (Figure 1).

### 3.2. RR Downregulated UVB-Induced ROS Production in NHDF Cells

**Figure 2:**
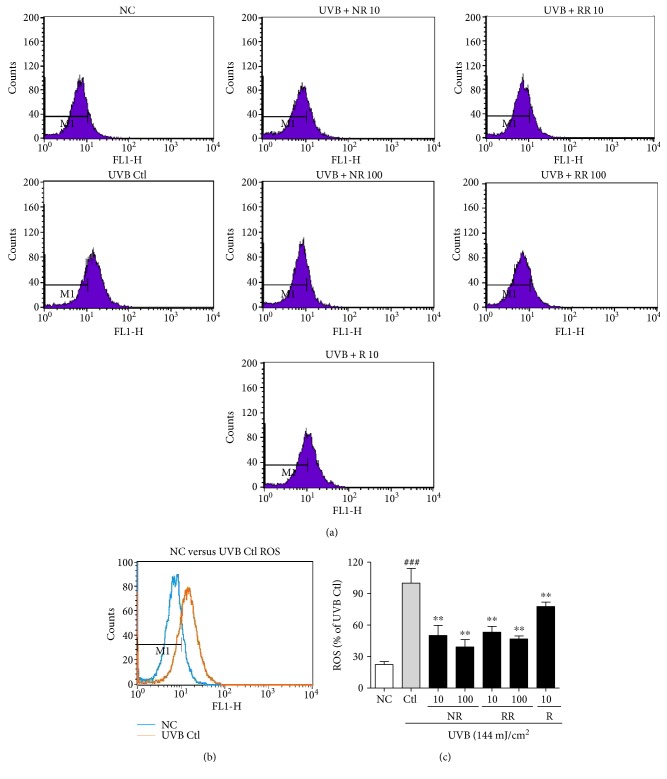
The levels of ROS in NHDFs treated as indicated for 24 h were measured using flow cytometry with DCFH-DA dye. The number of cells is plotted against the dichlorofluorescein fluorescence detected by the FL-1 channel (a). The relative ROS production of the cells is shown in each histogram (b). Values are mean ± SEM. The labels # and ∗ indicate significant differences (*p* < 0.05) when compared with the normal control and UV (+) control, respectively. ^###^*p* < 0.001 versus the normal control, ^∗∗^*p* < 0.01 versus the UVB-irradiated control. NC is normal control, Ctl is UVB control, NR is normal rice, RR is resveratrol-enriched rice, and R is resveratrol. NR and RR were treated in *μ*g/mL, and R was treated in *μ*M.

ROS are the major toxic substances generated by UVB exposure in the skin and dermal fibroblast cells. To measure ROS production in NHDFs, we treated cells with UVB and the samples for 24 h. The change in intracellular ROS compared with the nonirradiated controls was determined using 2′,7′-dichlorofluorescein diacetate (DCF-DA), which is oxidized by ROS in cells to DCF. The cells were stained with 30 *μ*M of DCF-DA and incubated for 30 min, after which the fluorescence level was measured. The UVB-induced ROS production in dermal fibroblast cells was significantly reduced following treatment with NR, RR, and R. RR demonstrated a greater reduction in ROS production at concentrations of 10 and 100 *μ*g/mL than NR or R alone. While all of the samples were capable of reducing UVB-induced ROS production, RR was found to be the most effective one (Figure 2).

### 3.3. NR, RR, and R Control the Level of MMP-1, TGF-*β*, and PIP-1 in NHDF Cells

**Figure 3:**
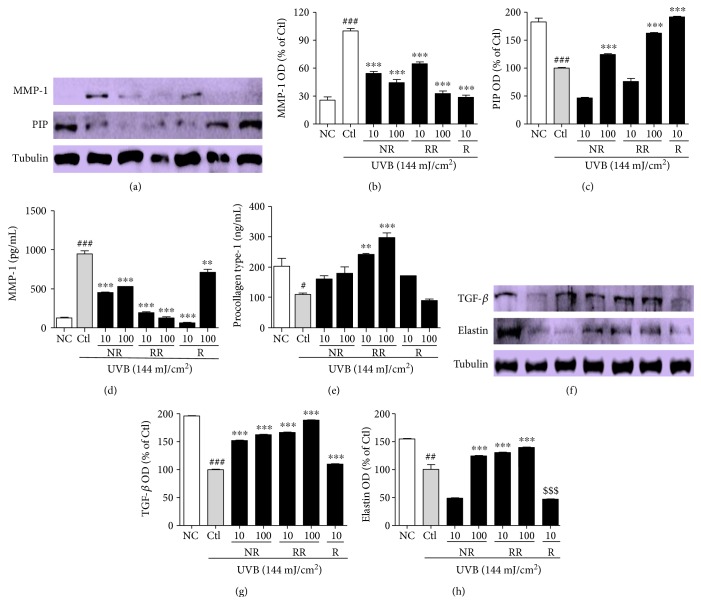
UVB-MMP1-mediated protein expression and the secretion of MMP-1 and PIP were measured using Western blot analysis and an ELISA kit, respectively. NHDF cells were incubated for 72 h with or without UVB exposure and treated with or without NR, RR, and R. (a) MMP-1 and PIP expression, accompanied by the corresponding (b, c) densitometric analysis results (d, e) and the quantities MMP-1 and PIP secreted into the treated medium supernatant. (f) TGF-*β* and elastin expression, accompanied by the corresponding (g, h) densitometric analysis results, taking the UVB-treated control as 100%. All data are presented as the mean ± SEM of three independent experiments. ^#^*p* < 0.05, ^##^*p* < 0.01, ^###^*p* < 0.001 versus the NC and ^∗∗^*p* < 0.01, ^∗∗∗^*p* < 0.001 versus the UVB control. NC is normal control, Ctl is UVB control, NR is normal rice, RR is resveratrol-enriched rice, and R is resveratrol. ^$$$^*p* < 0.001 versus UVB control represents the inhibition of elastin expression by resveratrol treatment. NR and RR were treated in *μ*g/mL, and R was treated in *μ*M.

To evaluate the effects of the samples on MMP-1 and PIP production in UVB-exposed NHDF cells, the levels of protein expression and MMP-1 and PIP-1 secretion were measured by using Western blotting and an ELISA, respectively (Figures 3(a), 3(b), 3(c), 3(d), and 3(e)). According to both the protein expression measurements and secreted protein assay, NR, RR, and R all reduced UVB-induced MMP-1 production and increased PIP production. The RR-mediated downregulation of MMP-1 and upregulation of PIP appear to have been caused by the additive effect of NR and R. This is because, despite R showing the most potent activity in reducing MMP-1 and increasing PIP levels (even at only 10 *μ*g/mL), the activity of RR seems to be better than that of NR and R alone in terms of the levels of secreted MMP-1 and PIP when tested using the ELISA kit. NR, RR, and R play significant roles in reducing MMP-1, but RR exhibits significantly greater (and concentration-dependent) activity against UVB-induced MMP-1 production. Only RR demonstrated the ability to increase PIP production almost two- and threefold at concentrations of 10 and 100 *μ*g/mL, respectively, in comparison with the UVB-treated control group, whereas NR and R were unable to affect a significant increase. This result stimulated our interest in elucidating the mechanism by which RR increases PIP levels to this extent. We therefore evaluated the protein expression of TGF-*β*, as the TGF-*β*/Smad pathway is a major pathway controlling PIP production. We found that RR significantly induced TGF-*β* protein expression in UVB-irradiated NHDF cells and thereby stimulated PIP production. This has the concomitant effect of preventing UVB-induced skin wrinkle formation, as upregulating TGF-*β* and PIP levels also results in increased elastin production. Similar result was obtained here in the RR and UVB-treated group. RR increased elastin production to a greater extent than NR and R alone (Figures 3(f), 3(g), and 3(h)). This result suggests that RR can protect NHDF cells against UVB-ROS-MMP-1-induced skin aging, particularly skin wrinkle formation.

### 3.4. RR Protects Human Reconstructed Skin Tissue against UVB-Induced Toxicity

**Figure 4:**
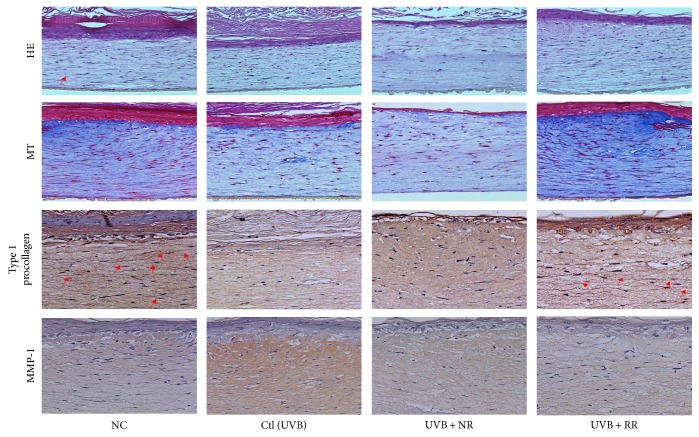
Photomicrographs of Masson's trichrome-stained sections and PIP-1 and MMP-1 production in reconstructed skin after treatment with UVB and samples (NR, RR, and R) at a concentration of 1% (*w/v*). NC is normal control, Ctl is UVB control, NR is normal rice, RR is resveratrol-enriched rice, and R is resveratrol. NR and RR were treated in *μ*g/mL, and R was treated in *μ*M.

To investigate the histological effects of RR on photoaging, UVB-exposed reconstructed human skin tissue was stained with H&E and MT. According to the H&E staining results, NR, RR, and R had no toxicity, although NR showed a mildly toxic effect in the epidermal layer. Staining with MT revealed the disruption and decomposition of collagen fibers in skin tissues exposed to UVB and that RR treatment in UVB-exposed skin tissue increased the abundance and density of collagen fibers (Figure 4). This indicates that RR protects skin tissue against UVB-induced collagen degradation in reconstructed human skin tissue. Furthermore, to investigate the effects of NR, RR, and R on type I procollagen and MMP-1 expression in reconstructed human skin tissue, we carried out immunohistochemical analysis on the sections. After UVB irradiation, the expression of MMP-1 increased and that of PIP-1 decreased. This effect was reversed with NR, RR, and R treatments especially for 10 *μ*g/mL for all samples and 10 *μ*g/mL for NR and RR samples, without any toxicity. Among these treatments, RR demonstrated the greatest efficacy (Figure 4). Hence, RR increased the expression of type I procollagen and decreased that of MMP-1 in reconstructed human skin, with the effect of protecting against UVB-induced skin aging or wrinkle formation.

### 3.5. RR Regulates the MAPK and AP-1-Mediated Signaling and Transcription in UVB-Irradiated NHDF Cells

**Figure 5:**
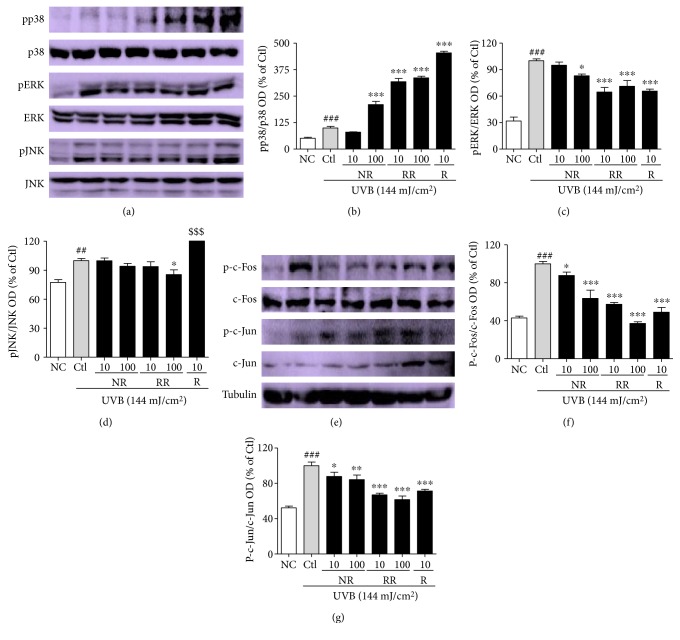
Protein expression levels of MAPKs and AP-1. (a) MAPK signal intensities from multiple experiments. (b, c, d) Bar graphs represent quantitative densitometric results of upper bands. (e) AP-1 protein intensities from multiple experiments. (f, g) Bar graphs represent quantitative densitometric results of upper bands. All data are presented as the mean ± SEM of three independent experiments. ^##^*p* < 0.01, ^###^*p* < 0.001 versus the NC and ^∗^*p* < 0.05, ^∗∗^*p* < 0.01, ^∗∗∗^*p* < 0.001 versus the UVB-irradiated control. NC is normal control, Ctl is UVB control, NR is normal rice, RR is resveratrol-enriched rice, and R is resveratrol. ^$$$^*p* < 0.001 versus UVB control represents the resveratrol-induced pJNK expression. NR and RR were treated in *μ*g/mL, and R was treated in *μ*M.

Inflammation-mediated skin aging is a major factor in UVB-induced skin aging and therefore a good target for controlling photoaging in dermal fibroblasts and skin more generally. Inflammation-mediated skin aging is initiated by the huge production of ROS that arises from UVB-induced oxidative stress in the skin. The excessive ROS production brought about by UVB activates MAPK signaling proteins to induce the AP-1- and NF-*κ*B-mediated transcription and translation of inflammatory proteins. To reproduce these conditions, we treated NHDF cells with UVB and the samples for 3 h, and the MAPK signaling proteins were evaluated. RR exhibited additive effects in this case, with NR, RR, and R downregulating the protein expression of pERK and pJNK but upregulating p38 expression (Figures 5(a), 5(b), 5(c), and 5(d)). Additionally, NR, RR, and R significantly downregulated p-c-Fos and p-c-Jun, which indicates that they inhibited the AP-1-mediated transcription of the inflammatory proteins responsible for inflammaging (Figures 5(e), 5(f), and 5(g)).

### 3.6. RR Inhibits the Inflammatory Cascades in UVB-Treated Dermal Fibroblast Cells Preventing Inflammaging

**Figure 6:**
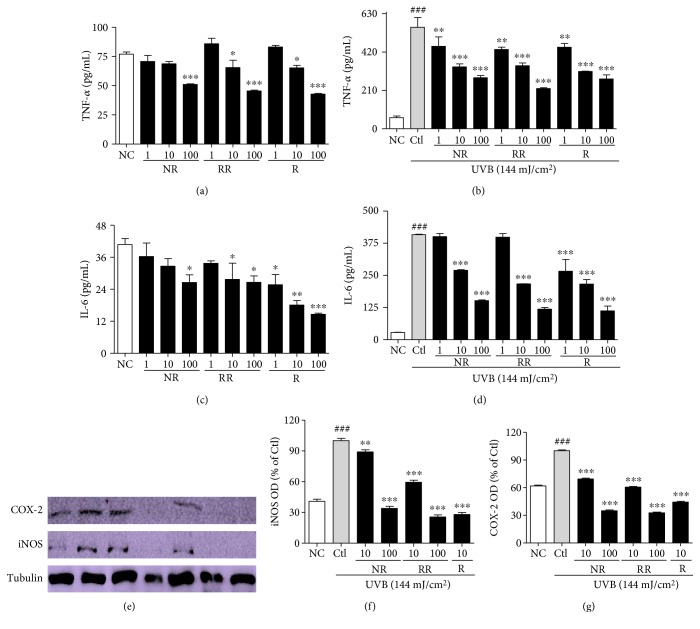
Proinflammatory cytokines (TNF-*α* and IL-6) secretion and inflammatory markers (iNOS and COX-2) expression. NHDF cells were treated with or without UVB (144 mJ/cm^2^) and with NR, RR, and R for 72 h. Proinflammatory cytokines were measured in cell supernatant using an ELISA kit, and protein expression of iNOS and COX-2 was measured using Western blot analysis. (a, b) TNF-*α* secretion in NHDF cells without UVB-conditioned medium and with UVB-conditioned medium. (c, d) IL-6 secretion in NHDF cells without UVB-conditioned medium and with UVB-conditioned medium. (e) iNOS and COX-2 protein expression from multiple experiments. (f, g) Bar graphs represent quantitative densitometric results of upper bands. All data are presented as the mean ± SEM of three independent experiments. ^###^*p* < 0.001 versus the NC and ^∗^*p* < 0.05, ^∗∗^*p* < 0.01, ^∗∗∗^*p* < 0.001 versus the UVB-irradiated control. NC is normal control, Ctl is UVB control, NR is normal rice, RR is resveratrol-enriched rice, and R is resveratrol. NR and RR were treated in *μ*g/mL, and R was treated in *μ*M.

AP-1, activated by MAPK signaling, induces the transcription and translation of inflammatory proteins, which further aggravates inflammation and makes the skin and cells more prone to aging, as well as cancer. To evaluate the production of proinflammatory cytokines, we performed an ELISA using the cell supernatants, following treatment with or without UVB and the samples, and the iNOS and COX-2 protein expression in those cells was evaluated. NR, RR, and R affected the concentration-dependent downregulation of TNF-*α* and IL-6 production in the conditioned medium with or without UVB treatment (Figures 6(a), 6(b), 6(c), and 6(d)). NR, RR, and R appear equally capable of reducing TNF-*α* secretion in the cells without UVB treatment. However, RR is more potent in this regard than NR and R alone when UVB treatment is applied. R elicited a marked reduction in IL-6 in both cases, but RR seems to be equally capable of reducing IL-6 levels in the cells treated with UVB. As R is a pure compound and NR and RR are merely extracts, we can clearly infer that the similarity in the efficacy of RR and R indicates the higher potency of RR in protecting cells against UVB-induced skin aging. Additionally, NR, RR, and R significantly downregulated the expression of iNOS and COX-2 in the dermal fibroblast cells (Figures 6(e), 6(f), and 6(g)). When applied at the higher concentration of 100 *μ*g/mL, the samples reduced the iNOS and COX-2 expression levels to below those observed in the normal control. The samples also caused the significant downregulation of iNOS and COX-2 when applied at 10 *μ*g/mL; RR exhibited greater potency than NR, but 10 *μ*g/mL of R was more potent than 100 *μ*g/mL of NR or RR.

### 3.7. RR Ameliorates UVB-Induced Apoptosis in Dermal Fibroblast Cells

**Figure 7:**
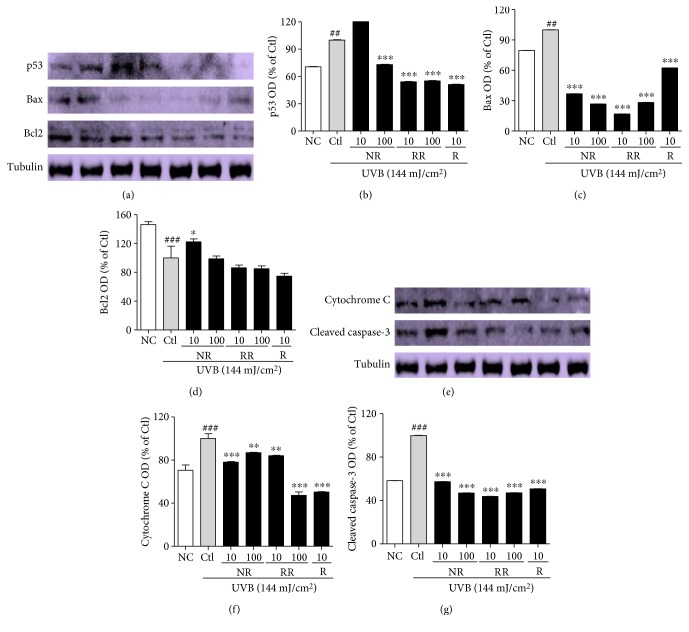
Apoptosis-induced skin aging and related protein expression in NHDF cells. NHDF cells were treated with or without UVB (144 mJ/cm^2^) and with NR, RR, and R for 72 h. Protein expression was measured using Western blot analysis. (a) p53, Bax, and Bcl2 protein expression from multiple experiments. (b, c, d) Bar graphs represent quantitative densitometric results of upper bands. (e) Cytosolic cytochrome C and cleaved caspase-3 protein expression from multiple experiments. (f, g) Bar graphs represent quantitative densitometric results of upper bands. Tubulin was used as a loading control. All data are presented as the mean ± SEM of three independent experiments. ^##^*p* < 0.01, ^###^*p* < 0.001 versus the NC and ^∗^*p* < 0.05, ^∗∗^*p* < 0.01, ^∗∗∗^*p* < 0.001 versus the UVB-irradiated control. NC is normal control, Ctl is UVB control, NR is normal rice, RR is resveratrol-enriched rice, and R is resveratrol. NR and RR were treated in *μ*g/mL, and R was treated in *μ*M.

**Figure 8:**
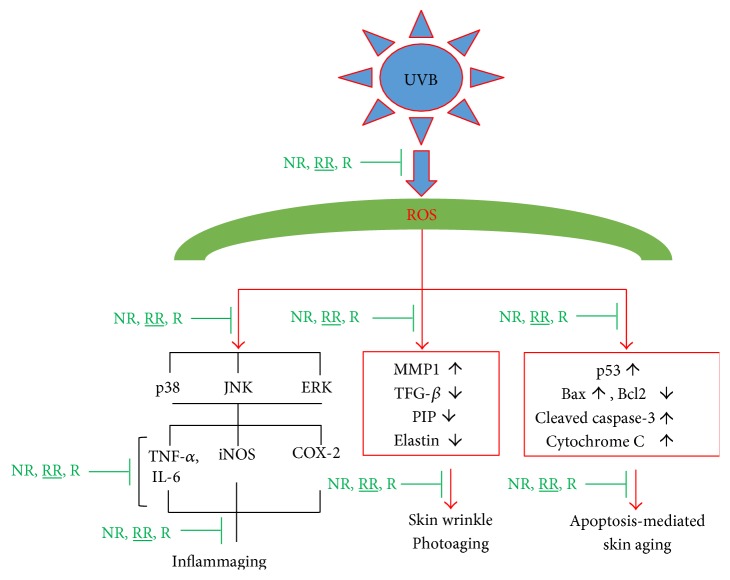
Scheme of the UVB-ROS-mediated skin aging and the protective role of resveratrol rice against its toxicity to prevent skin aging.

Apoptosis-induced skin aging can be induced by various aging processes, such as the excessive production of proinflammatory cytokines and inflammatory mediators, or the UVB-induced production of ROS. We observed that UVB-mediated ROS induced the expression of p53, Bax, Cytochrome C, and cleaved caspase-3 while reducing the expression of the antiapoptotic protein Bcl2 in dermal fibroblast. NR, RR, and R significantly reduced the production of proapoptotic proteins, such as p53, Bax, cleaved caspase-3, and cytochrome C, in UVB-treated dermal fibroblasts (Figure 7). RR and R were equally effective in downregulating the expression of p53, but RR reduced the expression of Bax, cleaved caspase-3, and cytochrome C more effectively. On the other hand, RR and R did not alter the expression of antiapoptotic protein Bcl2. In summary, RR can reduce the apoptosis arising from UVB-induced ROS production and inflammation but does not play a role in increasing antiapoptotic protein expression (Figure 7). Hence, RR promisingly inhibit the UVB-ROS mediated skin aging via various pathways such as MMP-1-mediated collagen degradation, inflammaging, and apoptosis-mediated aging as shown in (Figure 8).

## 4. Discussion

The changes in physical appearance brought on by aging, such as the development of wrinkles, can detrimentally affect the quality of life by impairing personal interactions, occupational functioning, and self-esteem. The prevention of skin aging and improvement of fine and coarse wrinkling in adults with minimal adverse effects are the main goals of skin care treatments [32, 33]. In this study, we examined the antiaging potential of normal rice (NR), resveratrol-enriched rice (RR), and resveratrol (R). Genetically engineered RR provided a similar biological effect to R, while avoiding its cellular toxicity even at 100 *μ*g/mL. RR was shown to control UVB-induced aging through downstream mechanisms in all the major pathways involved in skin aging and wrinkle formation. NR, RR, and R were found to control UVB-induced skin aging via downregulating oxidative stress-mediated aging, inflammation-induced skin aging, and apoptosis-mediated skin aging and wrinkle formation.

Photoaging is the hallmark of prolonged UV exposure. Exposing the skin to UVB activates oxidative stress in the normal rheostat of the dermis and epidermis or the respective cells that induce cell death, as well as altering cellular morphology [34–36]. In this study, we found that NR and RR do not have cellular toxicity, while 100 *μ*g/mL of R was toxic to NHDF cells. Although 10 *μ*g/mL of R was found not to be toxic in terms of cell viability, measured using an MTT assay, its toxicity was still evident through its adverse effects on cell morphology. These results demonstrate that, although it has promising biological activity, R has various toxic effects on the cells *in vitro*, which manifest as changes to NHDF cell morphology at the lower concentration (leading to shrunken cells, but not cell death) and cell death at the higher concentration. Furthermore, we found that NR, RR, and R promisingly reduced the UVB-induced production of reactive oxygen species (ROS) in normal human dermal fibroblasts. The UVB-induced production of ROS and free radicals upregulates the production of MMPs, particularly MMP-1, which is responsible for the degradation of the collagen network in tissue. This will result in the reduced secretion of TGF-*β* and subsequently procollagen, especially procollagen type II (PIP-II) [37, 38]. Furthermore, the downregulation of elastin and TGF-*β* in UVB-irradiated dermal fibroblast cells has been shown to further worsen the complications of the oxidative stress-induced aging process in the skin [39]. As elastin also plays an important role in the ECM of the dermis, its degradation leads to line and wrinkle formation in the skin. Therefore, agents that inhibit elastase activity are ideal candidates for the treatment or prevention of skin photoaging [40]. All of the samples described herein downregulated MMP-1 significantly, but the activity of RR appeared to be more potent than that of NR or R alone. Interestingly, a higher concentration of R (100 *μ*g/mL) resulted in increased MMP-1 production, whereas a lower concentration of R (10 *μ*g/mL) downregulated MMP-1 production more effectively than 100 *μ*g/mL of RR. This indicates that R is better able to reduce the level of MMP-1 than RR, even at a lower concentration. Additionally, a reduction in the protein expression for MMP-1 and increase in that for PIP were observed in the cells, as well as the treated cell supernatants. The effect of RR was found to be more promising than that of NR or R alone. UVB induced MMP-1 production and reduced the level of collagen in the dermis of the skin, but treatment with RR significantly decreased this toxicity and maintained homeostasis. Hence, the additive effect of NR and R, in the form of RR, was successfully demonstrated in NHDFs and reconstructed tissue. Only RR demonstrated an ability to increase PIP production almost two- and three-fold at doses of 10 and 100 *μ*g/mL, respectively, in comparison with the UVB-treated control group, whereas NR and R were unable to affect a significant increase. This result led us to investigate the mechanism by which RR so greatly increases PIP levels. We therefore measured the protein expression of TGF-*β*, as the TGF-*β*/Smad pathway is a major pathway controlling PIP production. We found that RR significantly induced TGF-*β* protein expression in UVB-irradiated NHDF cells and thereby stimulated PIP production. This can simultaneously prevent UVB-induced skin wrinkle formation, as upregulating TGF-*β* and PIP levels also results in increased elastin production. RR was shown to increase elastin production to a greater extent than NR and R alone. To confirm this *in vitro* finding, we conducted the same experiment in reconstructed tissue and obtained very similar results. Even in the reconstructed tissue, NR, RR, and R had no toxicity, although NR showed a mildly toxic effect in the epidermal layer. The disruption and decomposition of collagen fibers were observed in skin tissues exposed to UVB using Masson's trichrome (MT) staining, hematoxylin and eosin (H&E) staining, and staining for the determination of PIP and MMP-1. RR treatment increased the abundance and density of collagen fibers in UVB-exposed skin tissue. NR and RR treatment reduced the level of MMP-1 and increased that of type I procollagen in cells exposed to UVB. Of all the tested samples, RR most effectively increased the expression of type I procollagen and decreased that of MMP-1 in reconstructed human skin, thereby protecting against UVB-induced skin aging or wrinkle formation. These data collectively indicate that RR downregulates UVB-induced oxidative stress more potently than NR or R alone and therefore reduces its contribution to skin aging.

UVB irradiation induces ROS production. ROS-mediated oxidative stress activates MAPK signaling by increasing the phosphorylation of p38, JNK, and ERK (pp38, pJNK, and pERK) to induce inflammaging. Inflammaging is closely associated with many aging-associated diseases, such as Alzheimer's disease, as well as atherosclerosis, heart disease, type II diabetes, and cancer. One factor that exacerbates UVB-induced ROS-mediated inflammaging is immunosenescence [41]. Inflammaging is initiated after the activation of MAPK signaling, AP-1 (c-Fos and c-Jun) activation and the increased transcription of inflammatory mediators. Through the MAPK signaling pathways, AP-1 controls the expression of MMPs, especially MMP-1, MMP-2, and MMP-9, in inflammation-induced skin aging. Heterodimer complexes made between c-Jun and c-Fos, with various growth factors, cytokines, and UV exposure, can cause aggressive inflammation and skin aging [42]. In this study, we found that irradiating NHDF cells with UVB activates MAPK signaling, which activates the AP-1- and NF-*κ*B-mediated transcription of MMPs, proinflammatory cytokines, inflammatory mediators, and so forth, while simultaneously downregulating PIP, TGF-*β*, and elastin production [43]. Inflammatory mediators, such as iNOS, COX-2, and cytokines, as well as IL-6, IL-1*β*, and TNF-*α* produced by innate immune cells, will cause chronic inflammation and thus initiate inflammaging [44]. In this study, we demonstrated that treatment with NR, RR, and R can significantly modulate MAPK and AP-1 signaling, by inhibiting NF-*κ*B-mediated transcription. This was evidenced by the activation of PIP and the inhibition of TNF-*α*, IL-6, and MMP-1 in the treated dermal fibroblast cells. RR most effectively reduced TNF-*α* and IL-6 production in UVB-irradiated NHDF cells, but its ability to reduce iNOS and COX-2 levels, while still superior to that of NR, was inferior to that of R alone. This result further demonstrates the anti-inflammatory properties of these treatments and their great potential for protecting against UVB-induced inflammaging.

Oxidative stress and inflammaging together induce apoptosis, which is another key factor in skin aging, photoaging, wrinkling, and related disorders [45]. UVB-induced ROS cause further oxidative stress in the cellular environment, with the MAPK- and NF-*κ*B-mediated transcription of proinflammatory cytokines, particularly TNF-*α*, being the major cause of cell apoptosis and aging by apoptosis in the skin [46]. UVB, ROS, proinflammatory cytokines, and other toxins produced by UVB will induce the expression of apoptotic protein p53. This subsequently activates the expression of Bax, Bad, PUMA, and cleaved caspase-3, while simultaneously downregulating the expression of antiapoptotic proteins such as Bcl2 [47]. Activated cleaved caspase-3 translocates mitochondrial cytochrome C to the cytosol, which further induces apoptosis and contributes to skin aging by further activating factors that aggravate skin aging and wrinkle formation [19]. In this study, we confirmed that NR, RR, and R promisingly downregulate the levels of p53, Bax, cleaved caspase-3, and cytochrome C and that RR and R did not simultaneously alter the expression of the antiapoptotic protein Bcl2. RR downregulated the expression of Bax and cleaved caspase-3 more effectively than the other treatments and was as potent as R for the inhibition of p53 in the UVB-treated fibroblast cells. The RR-mediated reduction in p53 lowered the transcription of Bax and cleaved caspase-3. Bax and cleaved caspase-3 were consequently unable to translocate mitochondrial cytochrome C to the cytosol, thereby protecting fibroblasts from mitochondrial apoptosis and apoptosis-induced skin aging. In this way, and considering that RR (an extract) is generally at least as effective as R (a pure compound) or NR alone, we conclude that the pure compounds or standard compound in RR might have better synergistic biological activity than R alone for every considered aging pathway.

In conclusion, NR, R, and particularly RR have been shown to control MMP-1-mediated UVB-induced skin aging, apoptosis-induced skin aging, and inflammation-mediated complications called inflammaging in dermal fibroblasts. A schematic explanation for the UVB-ROS-mediated aging and the role of NR, RR, and R has been shown in Figure 8. In this way, our study has demonstrated the potential of RR as an antiaging product for the prevention of UVB-induced complications *in vitro* and ex vivo.
