# Corrigendum to “Resveratrol-Enriched Rice Attenuates UVB-ROS-Induced Skin Aging via Downregulation of Inflammatory Cascades”

**DOI:** 10.1155/2018/6052623

**Published:** 2018-02-20

**Authors:** Lalita Subedi, Taek Hwan Lee, Hussain Mustatab Wahedi, So-Hyeon Baek, Sun Yeou Kim

**Affiliations:** ^1^Laboratory of Pharmacognosy, College of Pharmacy and Gachon Institute of Pharmaceutical Sciences, Gachon University, Incheon 21936, Republic of Korea; ^2^College of Pharmacy, Yonsei University, No. 162-1, Songdo-dong, Yeonsu-gu, Incheon 406-840, Republic of Korea; ^3^Department of Biochemistry, Faculty of Biological Sciences, Quaid-i-Azam University, Islamabad, Pakistan; ^4^Department of Well-being Resources, Sunchon National University, Sunchon, Jeonnam 57922, Republic of Korea

In the article titled “Resveratrol-Enriched Rice Attenuates UVB-ROS-Induced Skin Aging via Downregulation of Inflammatory Cascades” [1], the affiliation of the fourth author was incorrect. The corrected authors' list and affiliations are shown above.
